# Continuity, change and ‘living well’ for older people with dementia: longitudinal qualitative findings from the IDEAL cohort study

**DOI:** 10.1017/S0144686X24000333

**Published:** 2025-04-04

**Authors:** Sally Stapley, Claire Pentecost, Alex Hillman, Ian Rees Jones, Robin Morris, Catherine Quinn, Madhumathi Ravi, Jeanette Thom, Linda Clare

**Affiliations:** 1 REACH: The Centre for Research in Ageing and Cognitive Health, University of Exeter Medical School, Exeter, UK; 2 Department of Criminology, Sociology, and Social Policy in the School of Social Sciences, Swansea University, Swansea, UK; 3 WISERD – Wales Institute of Social and Economic Research and Data, Cardiff University, Cardiff, UK; 4 Department of Psychology, Institute of Psychiatry, Psychology and Neuroscience, King's College London, London, UK; 5 Centre for Applied Dementia Studies, University of Bradford, Bradford, UK; 6 Faculty of Medicine and Health, The University of Sydney, Sydney, Australia; 7 NIHR Applied Research Collaboration South-West Peninsula, University of Exeter, Exeter, UK

**Keywords:** Alzheimer’s, Living Well, Quality of Life, Social Relationships, Occupation, Qualitative, Longitudinal

## Abstract

‘Living well’ is an important concept across national dementia strategies, where qualitative research has contributed to understandings of living well for people with dementia. Longitudinal, qualitative approaches are fewer but can explore potential changes in accounts of living well, psychological coping and adaptation to dementia, and if or how people with dementia maintain continuity in their lives. The aim of this longitudinal qualitative study was to gauge what is important for ‘living well’ with mild-to-moderate dementia and whether this changes over time in a group of older people with mild-to-moderate dementia living at home. Semi-structured, qualitative interviews with 20 people with dementia from the IDEAL cohort study were conducted in 2017 and again one year later and analysed using longitudinal thematic analyses. The overarching narrative was largely that of continuity and adaptation, with incremental not disruptive change. Continuing participation and meaningful occupation were important to maintain living well over time, where individuals pursued new as well as previous interests. As a key psychological coping strategy to support continuity in their lives, individuals emphasised their capabilities to maintain activities in spite of dementia, compartmentalising specific areas which had become more challenging. Maintaining social networks and accommodating changes in social relationships were also central to living well over time including managing the psychological impacts of changes in spousal relationships. People in the earlier stages of dementia emphasise continuity and their capabilities, reporting change over time only in certain aspects of their lives. However, small, incremental changes in their social relationships and opportunities for meaningful occupation may still afford key areas for supporting capability to ‘live well’.

## Introduction

Although the subjective experience of dementia is unique to the individual, the condition is characterised by progressive deterioration in cognitive and functional ability (WHO, 2022) as well as psychological, behavioural and physical symptoms (Alzheimer’s Society, 2022; Cohen-Mansfield, 2015). There is also considerable variability in rates of progression (Melis, Haaksma and Muniz-Terrera, 2019), so that individual disease trajectories are initially uncertain. Although dementia is a neurobiological condition, it should also be viewed within a psychosocial framework in which there are psychological responses to the condition within a social context (Bunn et al., 2012; Pratt and Wilkinson, 2003) including the shift to frame dementia as a disability (e.g. Shakespeare, Zeiling and Mittler, 2017; Cahill, 2022).

‘Living well’ with dementia is conceptualised as encompassing three inter-related constructs: quality of life, subjective well-being and life satisfaction (Clare et al., 2014). Adopted within several national dementia strategies and often associated with quality of life (Quinn et al., 2022), policy and practitioner perspectives have emphasised the multi-dimensional aspect of living well, with a focus on the significance of close personal relationships as well as social inclusion (Austin, O’Neill and Skevington, 2016). A systematic review showed that factors linked to relationships, social engagement and functional ability are associated with better quality of life (Martyr et al., 2018). In cross-sectional analyses in the IDEAL (Improving The Experience of Dementia and Enhancing Active Life) cohort study, psychological well-being was the most important predictor of capability to ‘live well’, with physical health, social circumstances and functional ability all contributing factors (Clare et al., 2019). Self-efficacy, optimism and self-esteem have also been identified as key psychological variables to live well with dementia (Lamont et al., 2020). Longitudinally, previous studies have reported that at group level, quality of life remains stable over time, although with individual variability (Livingston et al., 2008; Trigg et al., 2015). IDEAL cohort data identified, alongside groups with stable trajectories of good and poor quality of life, smaller groups with improving and declining trajectories (Clare et al., 2022a).

Although largely using cross-sectional methods, qualitative research has added to understandings of living well and quality of life for people with dementia. In their meta-synthesis of qualitative studies, O’Rourke et al. (2015) identified several critical concepts for quality of life in dementia including connectedness to family and relationships such as social interaction with others, wellness, and a sense of agency or purpose. Purposeful or meaningful activity affords people with dementia various benefits for quality of life (Han et al., 2016; Tierney and Beattie, 2020), with its importance for other groups also apparent; for example, those with neurodegenerative conditions such as multiple sclerosis and Parkinson’s disease (Desborough et al., 2020; Rafferty et al., 2021) and for older people in general (Smith et al., 2018). Other qualitative research has shown how people with dementia and their family members can have positive experiences and adapt to changing roles and relationships to live well with dementia (Wolverson, Clarke and Moniz-Cook, 2016; Colquhoun, Moses and Offord, 2019). However, a longitudinal qualitative approach focusing on living well may offer more nuanced insights into quality of life and individual experiences of living with dementia, and which of the various influences on living well reported retain the most significance for living well with dementia over time.

An additional strand to longitudinal research on living well for people with dementia is change over time and adaptation, and if or how they maintain continuity in their lives. ‘Change’ itself can be regarded as a transition which is dynamic and ongoing (Crafter and Maunder, 2012). For example, ‘change’ as a process is encompassed in continuity theory in ‘normal’ ageing, where ‘continuity’ is regarded as subjective and itself as a strategy for adaptation, where ‘continuity is not the opposite of change’ (Atchley, 1989, p.184). Framed by their ‘continuity maintenance’ approach to psychosocial adaptation in dementia (Lim and Song, 2019), these authors’ rapid realist review suggests how continuity may be sustained by maintaining personal and social identity, daily activities, and a familiar environment (Lim and Song, 2020). In dementia, multiple strategies for adaptation and maintaining continuity have been reported qualitatively, where emotional, cognitive and behavioural coping strategies include optimism, cognitive reappraisal and altering routines (Górska, Forsyth and Maciver, 2018). Again the importance of meaningful participation as a mechanism for supporting adaptation has been highlighted (Phinney, Chaudhury and O’Connor, 2007), where key contextual factors for participation include family and important others as well as formal services and local communities (Górska, Maciver and Forsyth, 2021). However, much of this qualitative research is cross-sectional only, so that longitudinal qualitative accounts are needed to explore potential shifts in accounts of psychological coping and adaptation to dementia, and how these may relate to living well with the condition over time. In relation to continuity and change, longitudinal qualitative analyses are of particular value (Hollstein, 2021; Neale, 2016; Saldaña, 2003).

Focusing on continuity, change and ‘living well’ over time, the current study uses longitudinal qualitative data from semi-structured interviews with older people with mild-to-moderate dementia to explore whether this can provide insights which address the gaps in available evidence. The aim of this qualitative component of the IDEAL cohort study was to gauge what is important for ‘living well’ with mild-to-moderate dementia, and to explore experiences of continuity, change and adaptation in participants’ accounts over time.

## Methodological Approach

### Study Design and Ethics

This qualitative study reports analyses of interview data gathered from people living with dementia who participated in the IDEAL cohort study. The aim of the qualitative component was to complement and enrich understanding gained from the IDEAL quantitative findings. HRA (Health Research Authority) was granted for this study. ‘Improving the experience of Dementia and Enhancing Active Life: living well with dementia. The IDEAL study’ was approved by the Wales Research Ethics Committee 5 (reference 13/WA/0405), and the Ethics Committee of the School of Psychology, Bangor University (reference 2014-11684). ‘Improving the experience of Dementia and Enhancing Active Life: a longitudinal perspective on living well with dementia. The IDEAL-2 study’ was approved by Wales Research Ethics Committee 5 (reference 18/WA/0111) and Scotland A Research Ethics Committee (reference 18/SS/0037). IDEAL and IDEAL-2 are registered with the UK Clinical Research Network (UKCRN), numbers 16593 and 37955. Because of the longitudinal aspect of the study, we were also cognisant of corresponding ethical issues such as temporality, ensuring anonymity when reporting due to the volume of data collected, and the need to counter the pre-existing researcher-researched relationship by reiterating participants’ right to refuse a second interview.

### Sampling and Recruitment

People living in the community with a clinical diagnosis of dementia and in the mild-to-moderate stages, as indicated by an MMSE (Mini-Mental State Examination) score of 15 or above (Folstein, Folstein and McHugh, 1975), were recruited to the IDEAL cohort study via UK Research Networks across 29 sites in England Scotland and Wales. Potential participants were identified through National Health Service memory services and other specialist clinics and the online UK Join Dementia Research portal, https://www.joindementiaresearch,nihr.uk. Recruitment was from 2014 – 2016, with participants followed at yearly or twice-yearly intervals for up to six time-points. Trained researchers had assessed participants’ capacity to consent using an ethically approved protocol for the cohort study, based on criteria set out in The Mental Capacity Act (2005), The Adults with Incapacity (Scotland) Act (2000) and The Mental Capacity Act Code of Practice (2005), with this code of practice based on four indicators of impairment: understanding, retaining information, weighing information, and the individual’s capacity to communicate a decision. All interviewees were able to provide informed consent at both interview timepoints, with ongoing consent monitoring (Dewing, 2008) also implemented during each of the two interviews. For the qualitative study, a maximum variation sample (Patton, 2014) was drawn from members of the IDEAL cohort study with diagnoses of Alzheimer’s, vascular or mixed dementia diagnosed at age 65 or above after Time 1 and Time 2 survey data had been collected.

We aimed to recruit 20 participants and to select interviewees based on evidence of positive or negative change in quality of life between these two time points (2014-2015) using a reliable change index calculated in an earlier study (Clare et al., 2014). In addition, the maximum variation sample considered gender, urban and rural location, and socio-economic background, based on income and previous occupations. Individuals with an MMSE score lower than 15 were ineligible to take part. To ensure a homogenous sample, people with rare dementia types or with young-onset dementia were not included in the qualitative sample at this stage because we had surmised that their understanding and experiences of living with dementia may differ from those with the more common forms of the condition. However, the diagnosis of one participant was changed to frontotemporal dementia after the interviews were conducted, and these data were retained in the analyses because her experiences were not dissimilar from those of other interviewees in the dataset.

### Longitudinal, Semi-Structured Interviews

The interview schedule for the first set of semi-structured interviews focused on ‘living well’ with dementia and reflection on any positive or negative changes due to the condition (see Supplementary Material 1). The topics were as follows: experience of change, home and activities, neighbourhood, social participation/outdoor activities, family and relationships, wider networks, services and support, and change and identity. In addition, sociograms, a visual representation of how people perceived their social networks, were used as a ‘talking tool’ to facilitate discussion of family, community connections and relationships with service providers (Ryan, Mulholland and Agoston, 2014). Although we had originally planned to analyse the sociograms quantitatively (Tubaro et al., 2016), it was apparent from the first interviews that this would have been of little value as most interviewees misinterpreted completion of the sociograms as needing to relate to geographical proximity of family and friends rather than to perceived closeness of such relationships or merged both approaches in their responses. Consequently, sociogram analyses were not completed, nor was sociogram completion repeated during the follow up interviews.

The interview schedule for the follow up, second set of semi-structured interviews focused on change in ‘living well’ since the first interview and whether interviewees felt any aspects of their lives had changed (see Supplementary Material 2). The interview schedule covered the same topics but with the addition of more social aspects of living well such as views on dementia-friendly communities. Interviewees’ first interview accounts were also used to prompt discussion. Sociogram completion was not repeated. Three of the second interviews were joint interviews with a family member but with the focus remaining on the experience of the person with dementia; this was because the participants had more difficulty engaging in the interview at this second time-point. During both sets of interviews, various potential psychosocial aspects of continuity, change and ‘living well’ were explored including neighbourhood, and wider networks, services and support, and prospective change in identity: at Time 1 (T1) in 2017, reflecting back over their experience of dementia to date, and at Time 2 (T2), one year later in 2018, reflecting on potential change since the first interview. First and follow up interviews were conducted by the same interviewer (AH), with first interviews lasting from 36 to 100 minutes, second interviews from 25 to 72 minutes, and joint second interviews up to 107 minutes.

### Longitudinal, Qualitative Analyses

Longitudinal thematic analyses were conducted (Derrington, 2019; Neale, 2020) and completed diachronically after both sets of data collection (Nevedal, Ayalon and Briller, 2019). To facilitate thorough familiarisation and data management, all interviews were summarised and represented in tabular format by SS, enabling an overview of the data as a whole. Through the familiarisation process, first and second interview content was contrasted, and key notes on content of potential analytic interest for changes in living well also noted in the table. In this way, focus was on the longitudinal aspect from the start of the analytic process. Thematic analysis is in six stages: familiarisation, coding, generating initial themes, reviewing themes, defining and naming themes, and writing up (Braun and Clarke, 2006; Clarke and Braun, 2021). The inductive, thematic analyses were facilitated using NVivo qualitative software (NVivo, 2020), where first interview codes were also applied to the second interviews but additional codes added as needed. Codes were then assimilated into initial themes. The thematic analysis process was adapted in accordance with the longitudinal approach, where inductive thematic analyses at the first and second time points were compared across the entire dataset.

To ensure that individual accounts had not become lost within the volume of data, themes were also compared narratively with individual cases (Thomson and Holland, 2003) to ensure an appropriate fit before themes were finalised. This enabled ‘conceptual scaffolding’ (Neale, 2016, p.110) and understandings of both group and individual experiences (Tuthill et al., 2020) in relation to living well and change over time. Rather than disregarding their contribution to the study, data from the three interviewees who were not re-interviewed contributed to development of the first interview codes. Familiarisation with a sample of the interviews by CP, prolonged engagement with the data and regular, ongoing discussion of the thematic analyses between SS and CP facilitated trustworthiness (Nowell et al., 2017). The final two themes and corresponding subthemes across both time-points were discussed with IDEAL team members and with our Patient and Public Involvement (PPI) group, known as the ALWAYs (Action on Living Well: Asking You) group, with the latter also concurring with our analyses. However, because of a large time gap between data collection and analyses due to team member changes, respondent validation with interviewees themselves was not conducted.

## Findings

Twenty people with dementia were recruited for the first qualitative interview in 2016, with 17 remaining in the study for the second interview one year later in 2017; two withdrew due to illness and hospitalisation, and one had died. The 20 interviewees ranged in age from 65 to 86 at the time of the first interview; 10 were male and 10 female, and all but three were living with a partner (see Table 1). Fifteen interviewees had Alzheimer’s, two vascular dementia, two mixed type (Alzheimer’s and vascular dementia), and one frontotemporal dementia.

Overall, people with dementia discussed little decline in their dementia symptoms over one year and therefore very little change, although decline over time was more apparent for some interviewees than others. Worsening co-morbid conditions such as arthritis and COPD (Chronic Obstructive Pulmonary Disorder) were considered more problematic by interviewees than living with dementia which was largely viewed with frustration only: “but other than that, no, life goes on” (Tom: T1). By their own comparison, individuals were able to live well with dementia but not necessarily with other health conditions which mostly they regarded as separate; only difficulties such as hearing problems seemed to compound the experience of dementia.

Importantly, emphasis was on continuity, where changes due to dementia were present but small and incremental one year on, although no less significant for the individual, and were not perceived as impacting all areas of people’s lives. In terms of supporting living well, how continuity was facilitated over time and change assimilated was linked to two key themes: ‘*Continuing participation and meaningful occupation: capability and compartmentalising’* and ‘*Maintaining social networks and accommodating changes in social relationships’*. One of our interviewees summarised the importance of both key themes for living well: “while ever you’re busy and you get on with people, you're enjoying life. But when one of them things stop, it goes to [the] bottom” (John: T1). In presenting these themes, accounts are considered across the dataset using pseudonyms but with particular focus on the longitudinal accounts of four interviewees: Jen, Max, Sarah and Tom.

### Continuing participation and meaningful occupation: capability and compartmentalising

This theme refers to the importance to maintaining living well over time by striving for continued engagement with interests and everyday tasks, even when these had become more challenging. A key psychological coping strategy to supporting continuity in their lives was individuals’ emphasis on their capabilities to maintain activities in spite of dementia, whilst circumscribing or compartmentalising specific areas which had become more difficult.

Interviewees emphasised “keeping the brain active” through maintaining hobbies as meaningful occupation or pursuing daily household routines which some perceived as preventing decline. Continuing participation in such activities was imperative for living well. Max did not let dementia deter him and spoke about his full day of activities including housework, gardening, and a new artistic interest he had pursued since his dementia diagnosis and now taught. By his second interview, Max had also taken up carpentry again:

“I don’t think it matters what the activity is… anything. Everybody can find a hobby… You must challenge yourself. Every day, you’ve got to get up and think, ‘what can I do?’” (Max: T2)

Striving for continued engagement and meaningful occupation was important for living well for other interviewees such as Jen who, since her first interview, was still able to pursue her previous activities and who had also started voluntary work:

“Um, living well, yes. Doing all the things I do: singing, art, playing badminton. I mean, I’m, I’m lucky. I can do all the things that I enjoy doing”. (Jen: T2)

It is interesting to observe that these interviewees’ accounts were not characterised by loss of activities but rather by continuation of past interests and even engagement with new ones, therein countering assumptions that dementia is always characterised by loss over time. For Max and Jen, only their sleep problems had worsened since their first interviews. By contrast, other interviewees who felt they were not doing something purposeful each day or who had given up past interests struggled to maintain living well. Fred missed work since retirement and needed to keep busy with activities every day: “once I lose them things, I get so miserable” (Fred: T1). For Grace, giving up dancing when local classes stopped, her only interest and when she had been happiest, was a huge loss for her:

“I used to dance two or three nights a week. I always got dressed up. Yeah, I miss that, like” (Grace: T2).

In addition to continued engagement with interests and everyday tasks for living well, a key psychological strategy was emphasis on capabilities rather than impairments due to dementia, where individuals compartmentalised areas of difficulty so that these did not dominate their lives. Jen preferred the term aphasia (wordfinding difficulties), saying that “dementia sounds like you’re going nuts” (Jen: T1). She discussed how her aphasia was only a problem when taking to people but that she could still pursue her interests such as working on her allotment:

“Yes, I mean there’s no problem with doing things. And when I’m doing things, I can forget that I haven’t got a good memory because it doesn’t matter anymore” (Jen: T2)

Compartmentalising, as a key psychological coping strategy, was apparent across several interviewees’ accounts, where interviewees emphasised how only some but not all aspects of their lives were affected by dementia:

“Er, well like I say everything else is fine, it’s just the lack of memory you know that’s what’s frustrating, that’s what is annoying as well. But apart from that, I can cope with everything you know doing the shopping, doing the housework and I can look after myself you know, shampoo my hair or whatever. I don’t have any problems like that. It’s just remembering things (Grace: T1)

“The easiest way is to go on walks or do the gardening or not meet people and then you're doing things you want to do and it's not affected. I mean I can still play codebreakers and things, it's not affecting my ability to do these things so … so long as I am doing those things, I am quite happy” (Susan: T1)

By contrast, some interviewees appeared to have lost confidence, with their accounts reflecting change and doubting ability, or they had given up interests and daily tasks entirely; for example, reading books or magazines, shopping and cooking:

“Um, and I feel inadequate, I suppose. Um, because I can’t do some of the things that I always used to do and never ever thought about. Um, so that’s… that’s my grievance with this”. (Pam: T2)

Some interviewees made temporal self-comparisons with past proficiencies:

“it saddens me a bit really that I realise I am not as mentally astute as I used to be…I think I probably rely on [my husband] quite a lot” (Sheila: T1).

Where interviewees had seemed to lose self-confidence, it was not always clear where this had stemmed from; for example, worsening symptoms or co-morbidities or perhaps from social expectations of what people with dementia are able to do. In a very few interviewees’ accounts, there also seemed to be physical representations of previous interests which were now being avoided, perhaps due to lack of self-motivation. One participant still had her art materials but had not painted for three years: “to be perfectly honest I've got awfully lazy, and I sit watching the television quite a bit” (Janet: T1). Another interviewee had a new printing press which had remained unused for six years, saying it was “almost too nice” to use, and there was “not the same impetus” to do his artwork (Bob: T1). By their second interviews, both Janet and Bob had still not reengaged with these past interests.

In our analyses, compartmentalisating was observed as a narrative device, where most interviewees described their experiences by emphasising their capabilities rather than impairments due to dementia; i.e. ‘I can still do X and Y, only Z is affected’. Compartmentalisating, as a psychological coping strategy, may serve a function in supporting adaptation to dementia by emphasising retained capabilities and avoiding cognitive challenge; e.g. not wanting to meet new people, or avoiding activities to prevent comparisons between current and past proficiencies. By compartmentalising, interviewees were striving to accommodate dementia within their everyday lives, at least during these early stages of their dementia trajectory. Here, there is also a dilemma for living well. If continued engagement with everyday tasks and past or new interests is important for living well over time, individuals who do not compartmentalise and avoid participation because they doubt their capabilities may be protecting themselves psychologically but at the same time be missing the benefits for living well sustaining such activities may afford.

### Maintaining social networks and accommodating changes in social relationships

The second theme important for continued living well concerns sustaining locally embedded social networks over time within supportive communities, particularly family or friends living close by, as well as managing the psychological impacts of changes in social relationships; for example, due to awareness of gradual shifts from a spousal relationship to more the dynamic of a partner as carer. The importance of strong family relationships for living well were apparent, which largely remained unchanged one year on, particularly where such social networks were embedded locally. For example, for some interviewees, their family was a short driving distance away and so visited regularly, like for Tom who saw his grandchildren every weekend; others whose family and friends lived at a distance were more socially isolated, even though they were in regular telephone contact with their families.

Like several of our interviewees, Tom and Jen had both lived in their respective homes for several decades and felt supported by longstanding neighbours and within the local community. Relationships with neighbours were also mutually supportive, where some interviewees helped their neighbours who were in poor health. Across both interview timepoints, most interviewees did not report experiencing stigma due to disclosing their diagnosis, although some spoke of misunderstandings about dementia. For example, by their second interviews, Jen and Sarah had been irritated by the dismissive reaction to their condition on disclosing this to other older people who, perhaps to ‘normalise’ dementia, had said that they also had memory problems:

“[they] wipe it away sort of thing” (Sarah: T2).

Maintaining social networks for living well over time was difficult, where interviewees were not seeing people as often due to their own physical health problems or those of their family and friends. With advancing age, interviewees also reported loss of family and friends due to bereavement.

Dementia could also have direct impacts on social interaction, for example, where individuals were managing aphasia and hence found it difficult to engage socially:

“I do sometimes feel left out for that reason”, “I’m not noticed” (Janet: T1).

By her second interview, Jen was concerned that eventually her aphasia may result in her getting to the point where she could no longer speak to anyone so she would be more dependent on her husband. Some interviewees like Sarah were at risk of loneliness due to both aphasia and hearing difficulties which Sarah said meant she did not have a lot of friends. Sarah lived with her daughter and daughter’s partner but was alone all day while they were at work. She was also dependent on her daughter to drive her to see friends. However, social networks and relationships were imperative to her such as meeting people and going out for a meal. By her second interview, social relationships were still central to her for living well, with her highlight now a weekly visit from a friend: “…it’s exactly the same, yeah. Yes, I love it when people come to see me” (Sarah: T2).

Likewise, Terry wanted to forge new friendships himself, having noticed his wife’s friendships and wanting something similar for himself:

“I haven’t got anyone saying you’re my friend” (Terry: T1).

By his second interview, Terry had now started going to watch football, where he had begun to develop more male friendships which had made a big difference to his life:

“I mean one of the big fear is…was that I had no one except my poor [wife] to talk to” (Terry: T2).

Therefore, even when people with dementia live with a partner, it cannot be assumed they do not feel lonely. Some interviewees attended dementia groups, enjoying the peer support this offered: “it’s the company, everybody in the same boat” (Pam: T2). Others, like Ria, disliked such groups, preferring to pay a formal carer to take her out and the companionship this afforded.

Maintaining social relationships was imperative for living well over time as well as managing the direct impacts of dementia such as aphasia on social interaction. Coping with changes in social, particularly spousal, relationships was also apparent for some interviewees; although most interview accounts suggested that the relationship with the family member had largely remained unaltered over time, with any physical or dementia-related decline accommodated within the spousal or filial relationship. Even the interviewee discussing her aggressive outbursts due to dementia described how these were circumscribed within her marriage:

“it’s a little part of our relationship that’s chipped out the whole lot” (Pam: T2).

Greater shifts in the relationship were apparent in some interviews, with one interviewee saying his wife now organised days out which was still the case by his second interview:

“where I was the leading partner, and now I’m not the leading partner” (Stan: T1).

Notably, by their second interview, two interviewees seemed to have become increasingly dependent on their spouses for their social interaction; one hoping her husband would retire so they could spend more time together (Pam: T2), and the other wanting her husband to stop going to football matches, to which he had interjected: “that’s the only thing I’ve got in life, other than looking after you” (Tina – husband: T2).

Some interviewees were aware that the relationship with their partner had shifted away from a spousal relationship towards more the dynamic of a partner as carer; although most avoided stating this explicitly, perhaps to avoid the psychological consequences such recognition might bring. Only one interviewee called his wife his “carer” or “nurse-carer” (Ian: T1 and T2), although individuals’ increasing care needs and declining physical health were apparent in some accounts, where some interviewees had been in respite care since their first interview. One interviewee needed more support by his second interview, previously having said he was “lost” without his wife and “terrified” of going anywhere without her: “I’m not allowed out on my own” (Ed: T1). Since his first interview, for Tom, there was a clear shift in perception of the relationship with his wife:

“Well, I think I feel for [my wife] at times, you know. With my brain not working properly sometimes, it gets frustrating for her, and I feel for her” (Tom: T2)

In comparison with his first interview, where Tom and his wife had just been on holiday abroad together, by his second, Tom’s wife was about to go on holiday without him, leaving their daughter to care for him: “it’ll give [my wife] a break from me” (Tom: T2). Therefore, in addition to maintaining social networks and relationships which were discussed explicitly as important for living well over time, managing the psychological impacts of accommodating shifts from spousal to carer relationships, where people with dementia are aware of these, is also of significance.

## Discussion

This longitudinal qualitative study has provided insights into the experiences of older people living with mild-to-moderate dementia over time. The overarching narrative was largely that of continuity and adaptation, with incremental not disruptive change. Changes, where present, were small, although no less significant for the person with dementia. Striving for continued engagement and meaningful occupation was important for living well over time, including the pursuit of new as well as past interests, countering assumptions that dementia is always conditioned by loss. Interviewees emphasised their capabilities, compartmentalising specific areas which had become more challenging as a key psychological strategy to support adaptation; conversely, others doubted their abilities including by making temporal self-comparisons with past proficiencies. In addition, sustaining locally embedded social networks over time was important for maintaining living well, such as family or friends continuing to live close by, as well as individuals managing the psychological impacts of changes in spousal relationships.

Both longitudinal themes resonate with previous research on the importance of meaningful occupation (Nyman and Szymcznska, 2016) and connectedness to family and relationships (O’Rourke et al. 2015) for living well and quality of life in dementia. Meaningful activities are central to continuity in the lives of people with dementia (Phinney, Chaudhury and O’Connor, 2007) as well as an important aspect of expressing and maintaining identity (Han et al., 2016; Stapley et al., 2023; Tierney and Beattie, 2020). In addition, meaningful occupation and valued activities are central to supporting agency in dementia (Chung, Ellis-Hill and Coleman, 2017). Our qualitative findings complement previous quantitative work from the IDEAL cohort study, from which our interviewees were drawn and what is important for living well with dementia (Quinn et al., 2022). However, our longitudinal qualitative work has highlighted what remains of importance over time for people with dementia, and how they adapt to the condition. Cross-sectional analyses of IDEAL quantitative data identified the relevance of adjustment to living with the condition and also continuity in sense of self (Clare et al., 2020; Clare et al., 2022b). Longitudinal, quantitative analyses from IDEAL have shown that quality of life scores were stable over time for 89% of IDEAL participants who were followed up after 12 and 24 months (Clare et al., 2022a). Supporting previous work (e.g. Pearce, Clare and Pistrang, 2002), our qualitative findings perhaps explain why quality of life remains stable, in that people with mild-to-moderate dementia may experience small but incremental changes over time, to which they are able to adapt. Compartmentalisation as the key psychological coping strategy enacted by our interviewees, where they emphasised how only some but not all aspects of their lives were affected by dementia, may be how individuals are able to adapt and maintain continuity in their lives (Atchley, 1989).

Moreover, the overarching narrative of continuity and incremental change over time is important to highlight, particularly for people with dementia themselves. Our interviewees’ accounts were characterised not by loss but by emphasising their capabilities, what they could still do, their new and past interests, where dementia was impacting some but not all aspects of their lives. Therefore, people with dementia can continue their lives, with agency and independence, accommodating the small, incremental changes which dementia may afford, at least in the earlier stages. This provides further support for ‘tragedy discourse’ counter-narratives focusing on agency and social citizenship (Bartlett and O’Connor, 2010), and the shift towards considering dementia a disability (e.g. Shakespeare, Zeiling and Mittler, 2017; Cahill, 2022). Notably, major changes may be absent in the earlier stages, and therefore understanding and monitoring the minutiae of cumulative change in people’s lives may be of greater import. For example, our work also highlighted the importance of maintaining social networks and relationships but also how small shifts in spousal to carer relationships may be apparent. Where the person with dementia becomes aware of changes in the spousal relationship and relational discontinuity (Riley et al., 2013; Riley, 2019), they may need support to adjust.

However, we are aware of some limitations of our work. Living well in our study is based on the accounts of older people with mild-to-moderate dementia specifically and largely with the more common forms of dementia; also our findings do not reflect the experiences of those with advanced dementia. Re-interviewing participants one year after their first interviews may have been an insufficient timeframe for dementia changes relevant to living well to become a significant concern. However, the notion of an optimal timeframe for re-interviewing is perhaps complicated by people with dementia adapting to their condition, as with our interviewees. A related point is that, although originally we attempted to incorporate positive or negative change within the sampling, changes in scores on the QoL-AD were relatively small, and therefore interviewees were considered as one group. Only in 5 cases was the change in either direction equal to or greater than the value of the reliable change index, which in fact turned out to be slightly greater in the IDEAL cohort than in the earlier study (Clare et al., 2022a). This in itself may support our finding of continuity and small, incremental changes in experiences of living well with dementia, although it is possible that participants with the greatest changes due to the condition may have been more likely to withdraw from the IDEAL cohort study. In future work, recruiting people with dementia and their family members who feel they are in crisis due to dementia may be difficult but of value.

Analysis was also carried out five years after the first data were collected due to factors including resourcing and the impact of the COVID-19 pandemic; hence the lead data analyst was not the same person who conducted the interviews. However, our findings regarding continuing participation and meaningful occupation are commensurate with qualitative work conducted during the pandemic, in which the importance of meaningful occupation for people with dementia and their identity was highlighted (Stapley et al., 2023). Use of sociograms may have inhibited interaction in the first interview rather than enhancing it, with some interviewees finding this approach difficult, hence this data was not used. In hindsight photo elicitation techniques or, where possible, ‘walking’ interviews (Thomson, 2012; Bates and Rhys-Taylor, 2017) may have been a more helpful approach particularly as interview topics included the home, neighbourhood and outdoor activities. In addition, there was minimal discussion by interviewees of health care, social care and community resources, and these were not explored in depth in the interviews. Participants were recruited to the IDEAL cohort on the basis of attendance at British memory clinics, and while the proportion of people from minority ethnic groups was consistent with British population estimates, numbers were small; while this explains why the interviewees in the current sample were all White British, it means that further work would be needed to ensure the perspectives of people from minority ethnic groups are represented. We are aware that including such individuals with dementia and their family members in research, for example, by improving recruitment strategies is imperative not only for their representation but to reduce inequalities in health and social care (Brijnath et al., 2022).

Nonetheless, our study has added to the corpus of longitudinal qualitative studies in dementia research. Largely situated within a narrative of continuity and adaptation, our work suggests that small, incremental changes in social relationships and opportunities for meaningful occupation may still afford key areas for support and intervention. Loneliness was apparent for some of our interviewees and was not always mitigated by existing partner relationships or attendance at dementia groups. Our findings serve as a reminder to health and social professionals of the importance of discussion around social supports outside the family carer relationship. Our work also emphasised opportunities for meaningful occupation, therein maintaining valued activities or facilitating new ones. Based on qualitative work in England and Wales with key stakeholders including general practitioners, a structured approach to the post-diagnostic annual dementia review has been suggested which includes discussion of psychological and emotional well-being and supporting relationships and meaningful activities (Bamford et al., 2021). However, such needs may be overlooked where people with dementia are also managing co-morbidities and physical health problems. In addition, activities of daily living may be more the focus in occupational therapy interventions in dementia (Bennett et al., 2019); although in personalised goal-oriented cognitive rehabilitation, there has been successful self-reported goal attainment including engaging in activities and personal projects (Clare et al., 2019). In our study, where interviewees seemed to have lost self-confidence in their valued activities, psychosocial interventions may have value to encourage engagement with and enjoyment of previous and new activities. This again serves to highlight the importance of resourcing such initiatives, although there is limited financial support for social care in the UK, where individuals mostly pay for it themselves (Henderson et al., 2019).

## Conclusion

In conclusion, this study is one of relatively few longitudinal qualitative projects in dementia research. The overarching narrative was largely that of continuity and adaptation, with incremental not disruptive change. In living well over time, most interviewees emphasised their capabilities and agency in pursuing valued activities, compartmentalising the impacts of dementia as a key psychological coping strategy for adaptation. Maintaining social networks over time was also important as well as managing the psychological impacts of changes in spousal relationships. However, understanding and monitoring incremental changes is still of value; in this way, smaller shifts in living well for people in the earlier stages of dementia are not missed. Therefore, for some people with mild-to-moderate dementia, facilitating social supports and opportunities for meaningful occupation may still afford key areas for interventions to promote living well, where both the preferences of the person with dementia and adequate resourcing of service provision are paramount.

## Supplementary Material

Supplementary material 1

Supplementary material 2

## Figures and Tables

**Table 1:** Participant Sociodemographic and Clinical Characteristics

No.	Gender	Age (at T1 survey interview in 2014)*	Ethnic background	Education	Urban/rural location	Dementia subtype	Time since diagnosis (at T1 survey interview in 2014)	MMSE (at T1 survey interview in 2014)*^1^
Ria	Female	64	White British	No qualifications	Urban	Alzheimer’s Disease	1-2 years	27
Tina	Female	76	White British	No qualifications	Rural	Mixed Alzheimer’s Disease/Vascular Dementia	1-2 years	22
Susan	Female	69	White British	University	Urban	Frontotemporal Dementia	Less than 1 year	30
June	Female	81	White British	University	Urban	Alzheimer’s Disease	1-2 years	23
Grace	Female	80	White British	School leaving certificate at age 16	Urban	Alzheimer’s Disease	Less than 1 year	26
Ian	Male	81	White British	University	Rural	Vascular Dementia	Not available	28
Jack	Male	70	White British	School leaving certificate at age 18	Urban	Alzheimer’s Disease	3-5 years	23
John	Male	78	White British	No qualifications	Rural	Vascular Dementia	3-5 years	23
Sheila	Female	66	White British	University	Urban	Alzheimer’s Disease	3-5 years	30
Fred	Male	76	White British	No qualifications	Urban	Alzheimer’s Disease	3-5 years	22
Janet	Female	74	White British	School leaving certificate at age 16	Rural	Alzheimer’s Disease	Less than 3 years	28
Bob	Male	74	White British	University	Rural	Alzheimer’s Disease	Less than 1 year	26
Pam	Female	65	White British	No qualifications	Rural	Alzheimer’s Disease	Less than 1 year	23
David	Male	69	White British	No qualifications	Rural	Mixed Alzheimer’s Disease/Vascular Dementia	Less than 1 year	24
Ed	Male	78	White Other	No qualifications	Urban	Alzheimer’s Disease	Less than 1 year	23
Terry	Male	78	White British	School leaving certificate at age 18	Rural	Alzheimer’s Disease	Not available	23
Pete	Male	69	White British	School leaving certificate at age 18	Urban	Alzheimer’s Disease	3-5 years	29
Stan	Male	84	White British	School leaving certificate at age 18	Urban	Alzheimer’s Disease	Less than 1 year	26
Una	Female	85	White British	University	Urban	Alzheimer’s Disease	1-2 years	25
Beth	Female	77	White British	University	Rural	Alzheimer’s Disease	Less than 1 year	25

* T1 qualitative interviews were conducted in 2017 and T2 qualitative interviews in 2018. No sociodemographic or clinical characteristics data including MMSE scores was recorded at these timepoints.

1 The Mini-Mental State Examination is a measure of cognitive function with a score range of 0-30 points.

## Data Availability

IDEAL data were deposited with the UK data archive in April 2020. Details of how to access the data can be found here: https://reshare.ukdataservice.ac.uk/854317/.
