# Supplementary material for: Continuity, change and ‘living well’ for older people with dementia: longitudinal qualitative findings from the IDEAL cohort study
Source: Ageing Soc. Author manuscript; Available in PMC 2025 Nov 1. (PMC7617875; doi:10.1017/S0144686X24000333)
Supplement: Supplementary material 1 [file EMS196323-supplement-Supplementary_material_1.docx]

**Supplementary Material 1: Interview Person with Dementia Version 2**

Introduction

This interview is interested in what helps you to live well, to feel good and enjoy life and what hinders you. We are interested in things that have may have changed in your life in recent months and years – for better or worse. We are interested in talking to you specifically because of your previous contact with the memory clinic.

1. Introductory questions/Experience of change

How are things for you at the moment? What’s happening for you at the moment that is important to your daily life?

- What’s a typical day like for you?
- Have you noticed any changes lately?
- How’s your general health would you say?
- Are you able to do the things you want to do?
- Are there things in your life (part of your daily routine, things you enjoy doing) you find more difficult to do?

1. Home & activities

Tell me about your home.

- Have you always lived here?
- Do you like living here?
- Do you like your house? What do you like about it? Are there things you would change about it? Where do you spend most of your time when you are in the house?
- Has this changed?

What do you like to do when you’re in the house?

(prompts- DIY, gardening, cooking, sewing, other crafts or handiwork - drawing, reading (being read to), listening to the radio, listening to music, watching TV, play games, crosswords, puzzles)?

Would you mind showing me your [cookbooks/puzzles/cross-words/pictures/drawings/music etc]?

- What is it you like about doing this?
- Is there anything that prevents you from doing it?
- Do you have any support from people/organisations that help you to carry on with your interests?

1. Neighbourhood

Do you like living in this neighbourhood? Why?

How do you feel when you go out in your local area?

Are there things you find difficult about being out of the house?

1. Social participation/Outside activities

Do you like spending time outside? Why is that?

Where do you go?

What do you like to do out of the house/home?

(Prompts – do you go to theatre, cinema, church/synagogue/mosque, shopping /exercise if not would you like to? Why, what is it that you enjoy/don’t enjoy? Has that always been the case or has this changed?)

**USE SOCIAL NETWORKS SOCIOGRAM ALONGSIDE QUESTIONS – AS PROMPTS**

1. Family & Relationships

*a). Marital/couple relationship (if married)*

Tell me about your husband/wife/partner? How did you meet?

Do you have any pictures you’d like to share (wedding day, when you met)?

- What is this of?
- How do you see yourself now compared to then?
- Has anything changed in your relationship more recently?

*b). Children & extended family*

Do you have children?

Do you have any pictures of them? Can I see them?

What do they do, where do they live, do they have children?

- Do you see them regularly? If not, are you in contact other ways? Do you talk on the phone, email, facetime?
- Do you talk to them about worries, concerns and difficulties? Do they ask you about your life?

What about other family – siblings, nieces, nephews?

(use same prompts)

*c). Friends*

Who are your friends?

What sort of things do you do together/talk about?

1. Wider Networks, Services & Support

Do you still have connections to your work, colleagues/friends, interests, activities?

Are you a member of any groups or clubs (perhaps related to activities/interests discussed earlier, or to your previous employment)?

Do you get any help from anyone outside your family and friends?

(prompts/suggestions- doctors, care workers, social workers, nurses, occupational therapists, physios etc.)

- What do you get help for?
- What do you think about this person/organisation?
- How do you feel about receiving this help/care?
- Are there aspects of your life where you need more help? What are they?

1. Summary Section – Change & Identity

What does living well mean to you? What makes for a good day?

What is important to you in your life?

Have things in your life changed from how it used to be in the past?

Do you think you’ve changed as a person?

Do you approach life differently?

Do people in your life behave differently around you? Do you think people think differently about you?

What are the aspects of your life that you are most happy with?

Thanks, is there anything else you would like to tell me?

END
