# Supplementary material for: Continuity, change and ‘living well’ for older people with dementia: longitudinal qualitative findings from the IDEAL cohort study
Source: Ageing Soc. Author manuscript; Available in PMC 2025 Nov 1. (PMC7617875; doi:10.1017/S0144686X24000333)
Supplement: Supplementary material 2 [file EMS196323-supplement-Supplementary_material_2.docx]

**Supplementary Material 2: Follow up Interview Person with Dementia**

Introduction

This interview is interested in how things have changed for you since we last met, particularly in relation to living well. We would like to know if aspects of your life have changed and whether that change makes it easier or more difficult to live the way you would like to, or to enjoy life.

1. Experience of change

How are things for you at the moment, what’s happening in your life at the moment that is important to you?

Have you noticed any particular changes?

- Has anything changed in your general health would you say?
- Are you able to do the things you want to do?
- Are there things in your life (part of your daily routine, things you enjoy doing) you find more difficult to do?

1. Home & activities – reflections on previous interview

In our last interview, you mentioned that you (enjoy living here; the house suits your needs; there are difficulties in doing what you need to do)

- Is this still the case, or has this changed? In what way?

Do you still enjoy doing the same kinds of activities at home? (things they mentioned in last interview - gardening, reading, listening to music)?

- Have you experienced any change to these activities? In what way?

Have you done anymore [artwork, writing, memoirs, scrapbook, etc. – something they mentioned in the previous interview] can I see them? Tell me how it’s been going?

- Has anything changed since we last met, in doing these activities? In what way?
- Do you still feel the same way about it as you did?
- Are your reasons for doing it the same as they were?

1. Neighbourhood

You mentioned in our previous conversation that you were (close to your neighbours, had good neighbours, didn’t know your neighbours). Is this still the case or has this changed since we last met?

Do you feel anything has changed about living in this area?

You mentioned about going shopping/for walks/to the local shop etc etc.

Are you still able to do these things? Is this still enjoyable?

Has anything changed that makes these activities more/less difficult?

1. Social participation/Outside activities

You mentioned in our last conversation about (gardening club, U3A, football, church etc.)

Is this something you are still involved with?

Are you involved in other kinds of groups/activities that are new since we last met?

Has your involvement in these groups/activities changed more recently? In what way?

Is there anything you find easier or more difficult about being part of this group/activity that is different to how it used to be?

**REFER TO THE ORIGINAL SOCIOGRAM THAT WAS FILLED OUT IN FIRST INTERVIEW – USE A DIFFERENT COLOUR PEN TO ANNOTATE THE SOCIOGRAM WITH ANY CHANGES OR ADDITIONS**

We talked last time about the people in your life, and we filled out this diagram together.

Talk through the sociogram with prompts:

Has there been any changes to your relationships you have with these people? In what way?

Do you feel anyone has become closer to you? Or has become more distant?

Are there any people that are important in your life that we didn’t talk about last time?

1. Family & Relationships

*a). Marital/couple relationship (if married)*

You talked about (the importance of your husband/wife; how the relationship has changed or stayed the same; ways in which your roles have changed etc)

Would you say this is still the case, or has anything a changed? In what way?

How have things been for you both since I saw you last?

*b). Children & extended family*

You talked about your relationship with your children

How are things with them/him/her since we met last?

Has anything changed? In what way?

Why do you think that is?

Have you had any family events/get togethers since we met, perhaps over Christmas? Do you have any photos you’d like to share?

How was that for you?

Is there anything different to those events now, compared to how they used to be?

C). *Friends*

You mentioned particular friend(s) when we spoke last time, how are things with them at the moment?

Has anything changed between you? In what way has it changed? Why?

1. Wider Networks, Services & Support

You mentioned last time that you were getting help from outside agencies/social support

Is this still happening? If not, what are the reasons for this?

Has this help increased since we met? Why is this?

How do you feel about this?

Do you feel you need more help/support? If yes, for what, from where?

OR

You mentioned when we spoke before that you don’t have a great deal of help from outside of family and friends, is that still the case or has that changed? If so how and why?

Do you feel you have the help/support you need? Or do you feel you need more? For what, from where?

1. Social aspects of living well

The current government is focused on helping people like yourself (with dementia/memory problems) to live well, do you think anything could be done at a community level to help you cope?

Do you think making shops and GP surgeries for example more dementia friendly (or more appropriate to the needs of older people/people with memory problems) would improve things for you?

1. Summary Section – Change & Identity

Is there anything that has happened to you since we last met which you feel has prevented you from living well? Can you tell me about it?

Is there anything that has happened to you since we last met which you feel has helped you to live well? Can you tell me about it?

You talked last time about the ways in which you feel you have changed as a person or your outlook has changed since your diagnosis.

Do you think you’re still changing? Do you see yourself differently?

When we spoke last time, you described living well or a good day as…..

Does this still reflect the things that are important to you, or has this changed? In what way?

Thanks very much. Is there anything else you would like to tell me before we finish?

END
